# Effects of *Hd2* in the presence of the photoperiod-insensitive functional allele of *Hd1* in rice

**DOI:** 10.1242/bio.021071

**Published:** 2016-10-26

**Authors:** Zhen-Hua Zhang, Li-Yong Cao, Jun-Yu Chen, Ying-Xin Zhang, Jie-Yun Zhuang, Shi-Hua Cheng

**Affiliations:** State Key Laboratory of Rice Biology and Chinese National Center for Rice Improvement, China National Rice Research Institute, Hangzhou 310006, China

**Keywords:** *Hd1*, *Hd2*, Heading date, Photoperiod-insensitivity, Rice, Yield traits

## Abstract

The role of photoperiod sensitivity (PS) of flowering genes have become well recognized in rice, whereas little attention has been drawn to the non-PS component of these genes, especially to their influence on gene-by-gene interactions. Rice populations in which the photoperiod-sensitive allele at *Hd1* has become insensitive to photoperiod but continued to affect heading date (HD) were used in this study to fine-map a quantitative trait locus (QTL) for HD and analyze its genetic relationship to *Hd1*. The QTL was delimitated to a 96.3-kb region on the distal end of the long arm of chromosome 7. Sequence comparison revealed that this QTL is identical to *Hd2*. In the near-isogenic line (NIL) populations analyzed, *Hd1* and *Hd2* were shown to be photoperiod insensitive and have pleiotropic effects for HD, plant height and yield traits. The two genes were found to largely act additively in regulating HD and yield traits. The results indicate that non-PS components of flowering genes involved in photoperiod response play an important role in controlling flowering time and grain yield in rice, which should allow breeders to better manipulate pleiotropic genes for balancing adaptability and high-yielding accumulation.

## Abstract (precis)

**Summary:** We show that photoperiod-insensitive components of alleles of *Hd1* and *Hd2* play an important role in balancing ecological adaptability and high-yield accumulation in rice.

## INTRODUCTION

Photoperiodic control of heading date (HD) is crucial for the seasonal and regional adaptation of rice (*Oryza sativa* L.). During the past decade, a large number of genes controlling HD in rice have been identified and their involvement in the molecular network for photoperiod response has been well characterized (Shrestha et al., 2014). Rice photoperiod sensitivity (PS) is mainly controlled by two pathways, the *Heading date 1* (*Hd1*) pathway which is conserved between rice and *Arabidopsis* (Yano et al., 2000; Kojima et al., 2002), and the *Early heading date 1* (*Ehd1*) pathway which is unique to rice (Doi et al., 2004). *Hd1*, a rice ortholog of *Arabidopsis CONSTANS*, exhibits dual functions on the flowering of rice depending on day length. It promotes heading under short-day (SD) conditions but is converted to repressing heading under long-day (LD) conditions through regulating the expression of *Heading date 3a* (*Hd3a*), a florigen gene in rice (Yano et al., 2000; Kojima et al., 2002; Tamaki et al., 2007). *Ehd1*, a rice gene that has no homolog in *Arabidopsis*, promotes flowering by activating the expression of *Hd3a* and another rice florigen gene, *Rice Flowering Locus T 1* (*RFT1*) (Doi et al., 2004; Komiya et al., 2008, 2009). *Ehd1* expression is downregulated by *Ghd7*/*Hd4* (Xue et al., 2008), *DTH8*/*Ghd8*/*Hd5* (Wei et al., 2010; Yan et al., 2011), *OsPRR37*/*Ghd7.1*/ *DTH7*/*Hd2* (Lin et al., 2000; Koo et al., 2013; Yan et al., 2013; Gao et al., 2014) and *EL1/Hd16* (Hori et al., 2013) but upregulated by *Ehd2* (Matsubara et al., 2008), *Ehd3* (Matsubara et al., 2011) and *Ehd4* (Gao et al., 2013). Several regulators of the *Ehd1* pathway, such as *Hd2*, *Hd5* and *Hd16*, require functional *Hd1* to repress flowering under LDs (Lin et al., 2000; Nonoue et al., 2008; Hori et al., 2013). Moreover, *Hd1* functions as an *Ehd1* repressor in the presence of functional *Ghd7* under LDs (Nonoue et al., 2008; Nemoto et al., 2016). The interactions between the two pathways form a complicated gene network for photoperiod response in rice.

HD is determined not only by PS, but also by the basic vegetative growth period (Vergara and Chang, 1985; Nishida et al., 2001). It has been reported that functional genes for photoperiod response in rice could play an additional role in regulating flowering under the optimal conditions of photoperiod and temperature (Nakagawa et al., 2005). For many of the rice genes involved in PS, the role of regulating flowering through photoperiod response has become well recognized, but little attention has been drawn to the effect of the non-PS component of these genes, especially their influence on the gene-by-gene interaction for HD and associated traits. Zhenshan 97 (ZS97) is an early-season rice variety carrying the functional PS allele *Se-1^u^* at the *Hd1* locus but has been shown to be photoperiod insensitive (Wei et al., 2012). As compared with Milyang 46 (MY46) carrying photoperiod-insensitive allele *Se-1^e^*, the ZS97 allele promotes heading under both natural short-day (NSD) and natural long-day (NLD) conditions, and meanwhile decreases plant height, spikelet number, grain number and grain yield (Zhang et al., 2012).

In the present study, fine-mapping of a quantitative trait locus (QTL) for HD and analysis of the genetic relationship between this QTL and *Hd1* were conducted using near isogenic lines (NILs) and NIL-F_2_ populations developed from the *indica* rice cross ZS97///ZS97/ZS97/MY46. The QTL was delimitated to a 96.3-kb region and found to be identical to *Hd2* based on the map position, genome annotation and coding sequence comparison. At the *Hd2* locus, the MY46 allele is functional and ZS97 allele is non-functional. In the NIL population analyzed, the functional *Hd1*^ZS97^ and *Hd2*^MY46^ alleles were shown to be photoperiod insensitive and acted additively to affect HD and yield traits.

## RESULTS

### Fine mapping of the *qHD7.2*

**Fig. 1.**
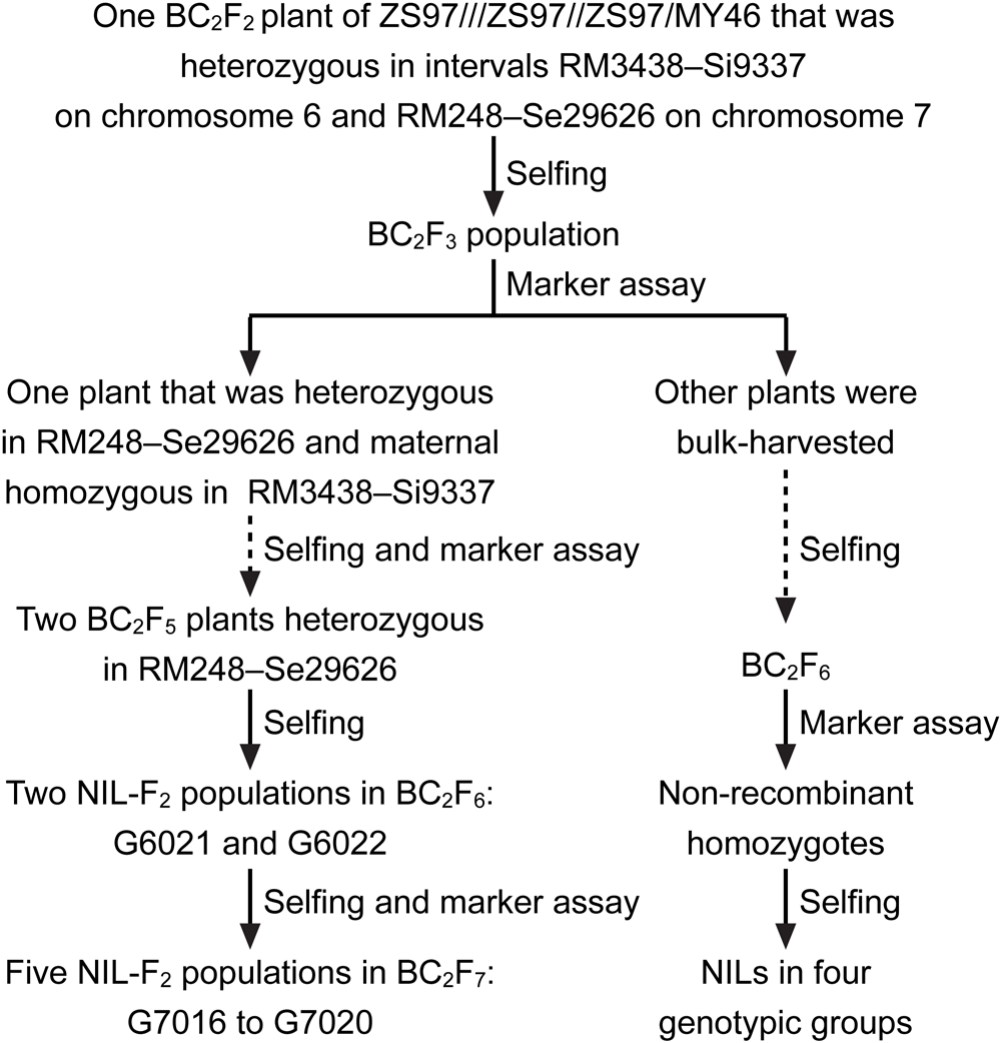
**Development of the rice populations.** The seven NIL-F2 populations were used for fine-mapping *qHD7.2*, and the NILs in four genotypic groups were used for analyzing the interaction between *Hd1* and *Hd2*.

**Fig. 2.**
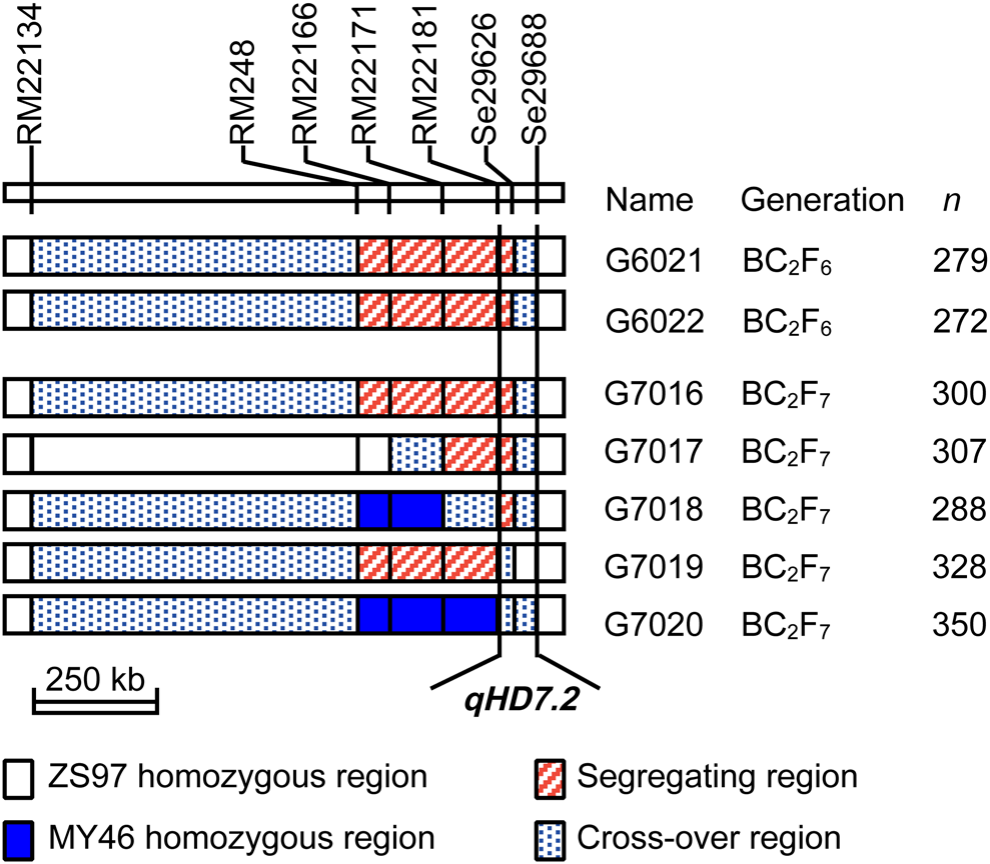
**Genotypic compositions of the seven NIL-F_2_ populations in the target region harboring *qHD7.2*.** These populations were used for fine-mapping *qHD7.2*. *n*, number of plants in each population.

In this study, *qHD7.2* was firstly validated using two NIL-F_2_ populations in the BC_2_F_6_ generation and then fine-mapped using five NIL-F_2_ populations in BC_2_F_7_. The two BC_2_F_6_ populations were progenies of two BC_2_F_5_ plants which were derived from a BC_2_F_2_ plant of an *indica* rice cross between the recurrent parent ZS97 and the donor parent MY46 (Fig. 1), respectively. They were segregated in interval RM248–Se29626 on the distal end of the long arm of chromosome 7 (Fig. 2) in which a QTL for HD, *qHD7.2*, was previously detected in the ZS97/MY46 recombinant inbred line (RIL) population (Zhang et al., 2011). In the genetic background tested with 138 polymorphic markers, the two BC_2_F_5_ plants were totally homozygous and had 87.68% identity to ZS97.

The two BC_2_F_6_ populations were grown under NLD conditions in Hangzhou, Zhejiang, China in 2010 (Table 1), with G6021 and G6022 consisting of 279 and 272 plants, respectively. In both populations, HD exhibited bimodal distributions with the ZS97 homozygotes flowering earlier than the MY46 homozygotes (Fig. S1). QTL analysis with marker data of RM248 and Se29626 showed that *qHD7.2* explained 72.03% and 80.40% of the phenotypic variance in G6021 and G6022, with the MY46 allele delaying heading by 5.70 days and 5.59 days, respectively (Table 2).

**Table 1.**
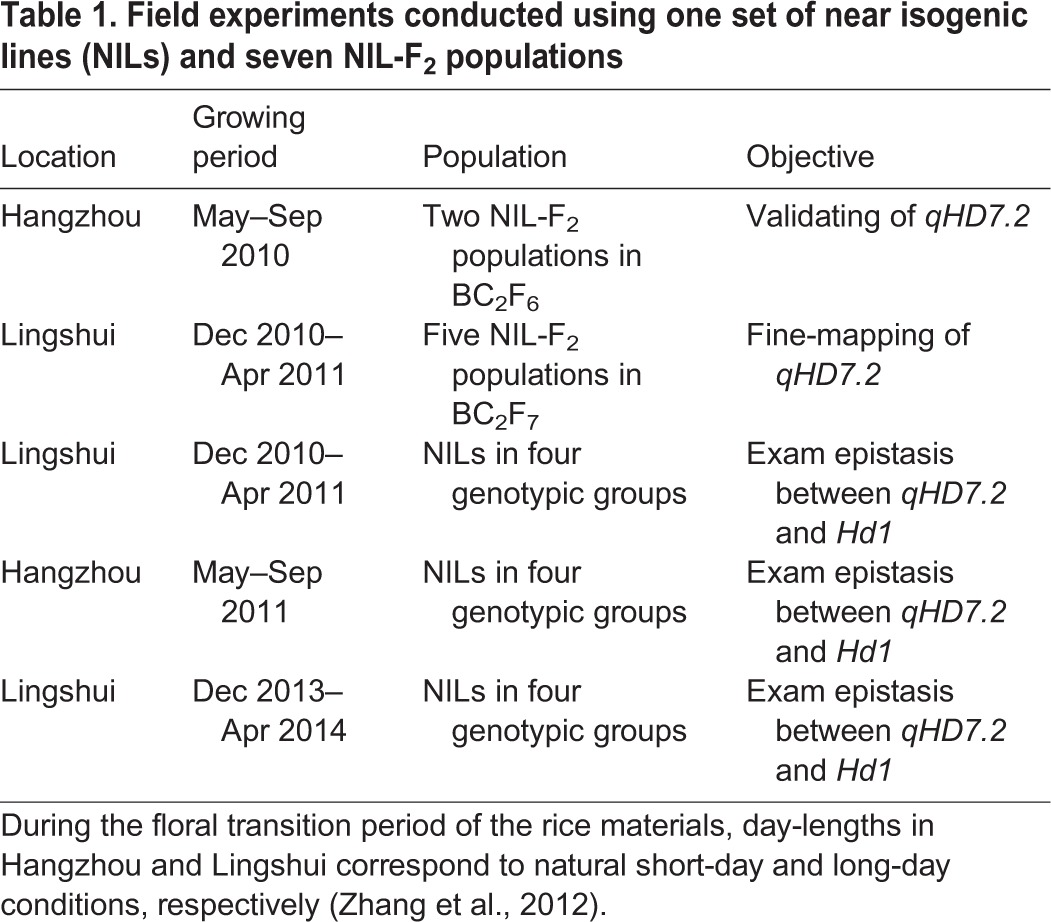
**Field experiments conducted using one set of near isogenic lines (NILs) and seven NIL-F_2_ populations**

**Table 2.**
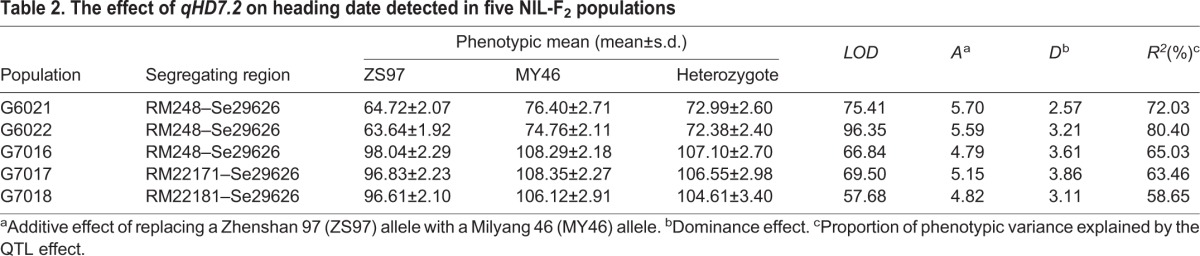
**The effect of *qHD7.2* on heading date detected in five NIL-F_2_ populations**

Five NIL-F_2_ populations in BC_2_F_7_ were produced to fine map the *qHD7.2*. They were segregated in the marker regions RM248–Se29626, RM22171–Se29626, RM22181–RM29626, RM248–RM22181 and Se29626, respectively (Fig. 2). These populations were grown under NSD conditions in Lingshui, Hainan, China from Dec 2010 to Apr 2011 (Table 1), with the number of plants tested in one population ranging from 288 to 350. HD showed bimodal distributions in three of the populations, G7016, G7017 and G7018, with the values ranging from 93 days to 105 days for the ZS97 homozygotes and from 102 days to 114 days for the MY46 homozygotes (Fig. S1). HD of the remaining two populations were much more uniform, with G7019 showing earlier heading ranging from 91 days to 105 days and G7020 having later heading from 104 days to 114 days (Fig. S1). These results indicated that *qHD7.2* was segregated in populations G7016, G7017 and G7018, while it was ZS97 and MY46 homozygous in G7019 and G7020, respectively. As shown in Fig. 2, the RM22181–Se29688 interval contained a segregating region and a cross-over region in populations G7016, G7017 and G7018, a ZS97 homozygous region and a cross-over region in G7019, and a cross-over region flanking the heterozygous marker Se29626 in G7020. The *qHD7.2* was therefore delimitated within the 96.3-kb genome region between RM22181 and Se29688. QTL analysis showed that *qHD7.2* explained 65.03%, 63.46% and 58.65% of the phenotypic variance in G7016, G7017 and G7018, with the MY46 allele delaying heading by 4.79 days, 5.15 days and 4.82 days, respectively (Table 2). This QTL was shown to be insensitive to photoperiod, as the effects detected are similar between the three BC_2_F_7_ populations grown in the NSD conditions in Lingshui and the two BC_2_F_6_ populations tested in the NLD conditions in Hangzhou.

Based on the MSU Rice Genome Annotation Project, 12 putative genes are located in the 96.3-kb region. One of them, LOC_Os07g49460, also known as *Hd2*/*Ghd7.1*/*DTH7*/*OsPRR37*, is involved in regulating rice heading date (Lin et al., 2000; Koo et al., 2013; Yan et al., 2013; Gao et al., 2014). Sequence comparison of *Hd2* coding sequences between the two parental lines was made (Fig. S2). ZS97 has an 8-bp deletion in the seventh exon which has been known to cause the loss of function of *Hd2* (Koo et al., 2013; Yan et al., 2013). Except for a C/T substitution at the S1773 site which resulted in a synonymous mutation G591G, the MY46 allele is identical to the strong functional allele *PRR37-1* reported by Koo et al. (2013) and Hap1 by Yan et al. (2013). Thus, *Hd2*/*Ghd7.1*/*DTH7*/*OsPRR37* is the gene responsible for the QTL *qHD7.2*, and we renamed *qHD7.2* as *Hd2*.

### Main and epistatic effects of *Hd1* and *Hd2* on heading date

**Fig. 3.**
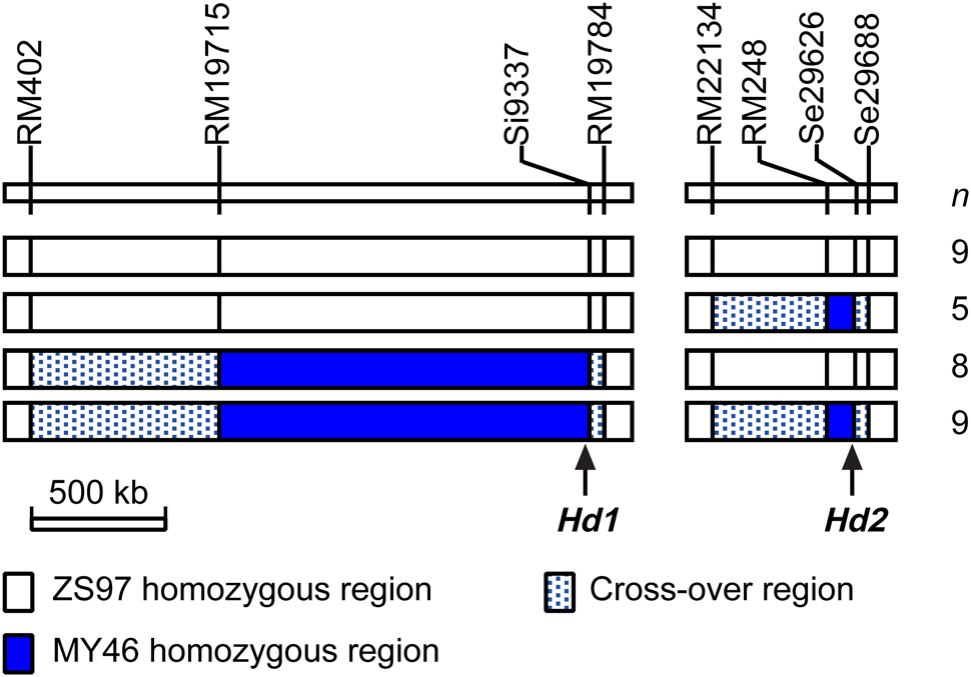
**Genotypic composition of the four NIL sets in the two target regions harboring *Hd1* and *Hd2*, respectively.** These populations were used for analyzing the interaction between *Hd1* and *Hd2*. *n*, number of near isogenic lines in each genotypic group.

The original BC_2_F_3_ population was segregated in both the RM3438–Si9337 region harboring *Hd1* and the RM248–Se29626 region harboring *Hd2* (Fig. 1). This population has been used to construct NILs for analyzing the effects of *Hd1* (Zhang et al., 2012). It was found that the functional *Hd1*^ZS97^ allele was insensitive to photoperiod in the genetic background of ZS97 but promoted heading by 4.64−6.14 days under NSD and NLD conditions. In the present study, one set of NILs comprising all the four homozygous genotypic combinations of *Hd1* and *Hd2* were developed and used to investigate the genetic relationship between the two genes. The NIL set consisted of nine lines of *Hd1*^ZS97^*Hd2*^ZS97^, five lines of *Hd1*^ZS97^*Hd2*^MY46^, eight lines of *Hd1*^MY46^*Hd2*^ZS97^, and nine lines of *Hd1*^MY46^*Hd2*^MY46^ (Fig. 3). They were tested in three trials, including two trials under NSD conditions in Lingshui and one trial under NLD conditions in Hangzhou (Table 1).

*Hd1* and *Hd2* showed a significant effect on HD in each of the three trials (Table 3). As compared with the ZS97 allele, the MY46 allele delayed heading at both loci and the effects estimated were a little higher for *Hd1* than for *Hd2* in all the trials. In the first trial conducted in Lingshui from Dec 2010 to Apr 2011 (11LS), the additive effects of *Hd1* and *Hd2* were 6.34 days and 5.41 days, explaining 44.26% and 31.29% of the phenotypic variances, respectively. In the trial conducted in Hangzhou from May to Sep 2011 (11HZ), the additive effects of *Hd1* and *Hd2* were 5.08 days and 4.88 days, explaining 37.82% and 38.55% of the phenotypic variances, respectively. In the second trial conducted in Lingshui from Dec 2013 to Apr 2014 (14LS), the additive effects of *Hd1* and *Hd2* were 6.64 days and 4.45 days, explaining 60.53% and 17.31% of the phenotypic variances, respectively.

**Table 3.**
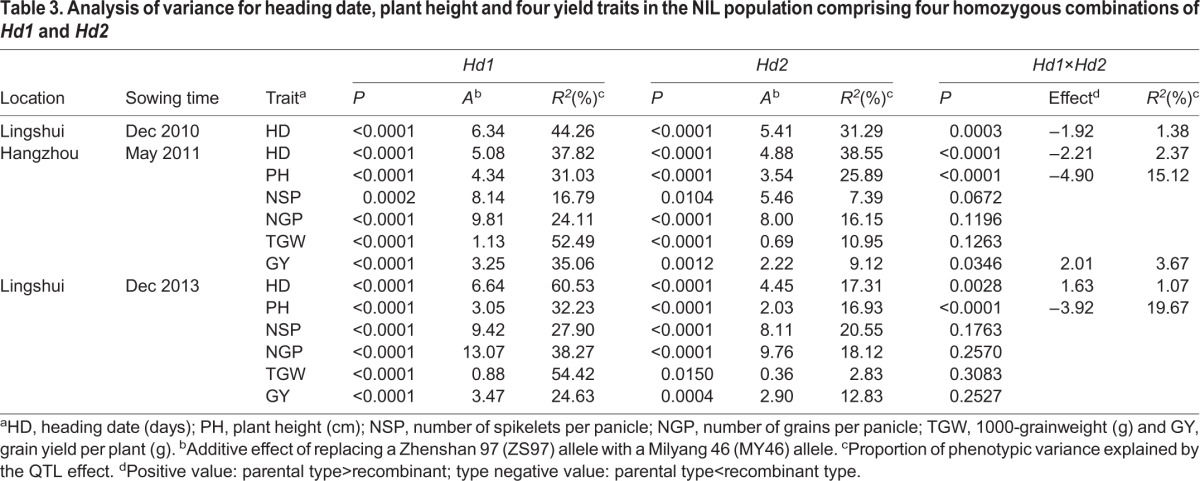
**Analysis of variance for heading date, plant height and four yield traits in the NIL population comprising four homozygous combinations of *Hd1* and *Hd2***

**Fig. 4.**
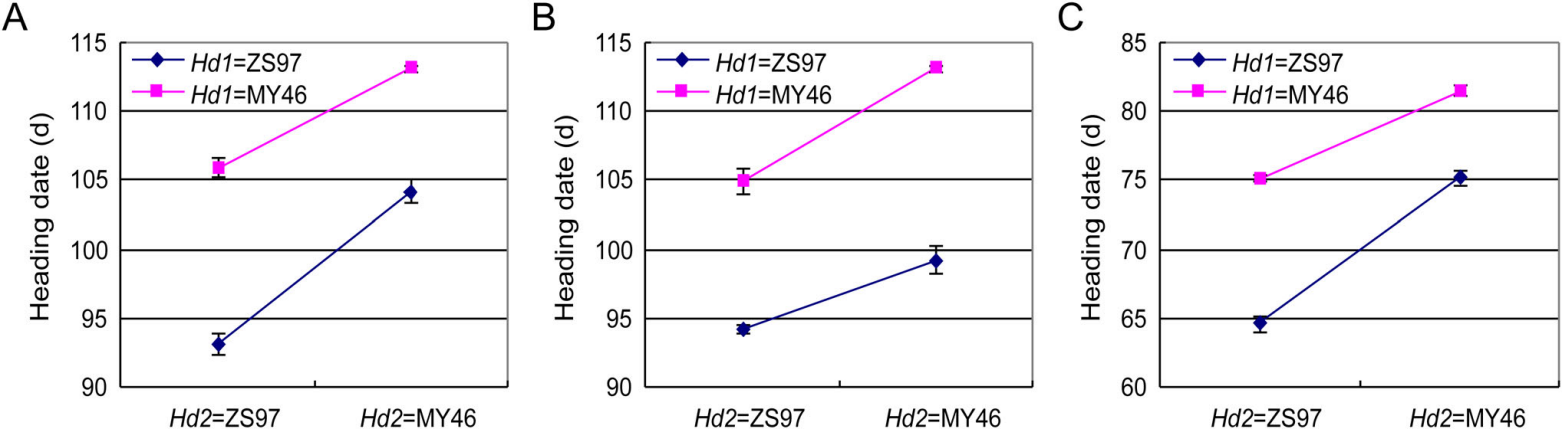
**Heading date of the four NIL sets.** (A) Under the natural short-day (NSD) conditions in Lingshui in the growing season of Dec 2010−Apr 2011. (B) Under the NSD conditions in Lingshui in the growing season of Dec 2013−Apr 2014. (C) Under the natural long-day (NLD) conditions in Hangzhou in the growing season of May−Sep in 2011. Data are represented as mean±s.e.m. Number of lines for NIL sets: *Hd1*^ZS97^*Hd2*^ZS97^, *n*=9; *Hd1*^ZS97^*Hd2*^MY46^, *n*=5; *Hd1*^MY46^*Hd2*^ZS97^, *n*=8; *Hd1*^MY46^*Hd2*^MY46^, *n*=9. Each line was tested in two replicates and eight plants per replicate were scored.

Significant digenic interaction between *Hd1* and *Hd2* was detected, but the effect was much smaller compared with the main effects of the two genes (Table 3). The functional *Hd1*^ZS97^ and *Hd2*^MY46^ alleles promoted and suppressed heading, respectively, no matter whether its counterpart was functional or non-functional and the NILs were grown under NSD or NLD conditions (Fig. 4). Compared with the *Hd1*^ZS97^*Hd2*^ZS97^ group, which had the earliest flowering time, replacement of the *Hd1*^ZS97^ by *Hd1*^MY46^ alone (the *Hd1*^MY46^*Hd2*^ZS97^ group) delayed heading for 12.80 days and 10.57 days in the 11LS and 11HZ trials under NSD and NLD conditions, respectively. Similarly, replacement of the *Hd2*^MY97^ by *Hd2*^ZS97^ alone (the *Hd1*^ZS97^*Hd2*^MY46^ group) delayed heading for 11.07 days and 10.65 days in the two trials, respectively. In occurrence of genotype replacement at both loci (the *Hd1*^MY46^*Hd2*^MY46^ group), heading was delayed by 20.04 days and 16.98 days in the 11LS and 11HZ trials, respectively. In these trials, genotype alternation at both the *Hd1* and *Hd2* loci had much stronger effect as compared with the effect due to genotype alternation at either locus although the cumulative effect was a little lower than the additive values of the two single-alternations. In the 14LS trial under NSD conditions, genotype replacement at both loci delayed heading for 18.90 days, which are a little higher than the summation of the 10.68 days and 4.96 days due to single replacement at *Hd1* and *Hd2*, respectively. Overall it could be concluded that *Hd1* and *Hd2* largely act additively in the NIL populations analyzed in this study.

### Main and epistatic effect of *Hd1* and *Hd2* on plant height and yield traits

Plant height (PH) and yield traits including the number of spikelets per panicle (NSP), the number of grains per panicle (NGP), 1000-grain weight (TGW) and grain yield per plant (GY) were scored for NILs tested in the 11HZ and 14LS trials. In both trials, *Hd1* and *Hd2* showed significant effects on all the five traits, though the effects of *Hd2* on NSP and TGW were marginal in the 11HZ (*P*=0.0189) and the 14LS trials (*P*=0.0150), respectively (Table 3). The enhancing alleles were derived from MY46 at both loci for all the traits, matching the allelic direction of the two genes on HD. In the 11HZ trial, the additive effect of *Hd1* and *Hd2* was 4.34 and 3.54 cm for PH, 8.14 and 5.46 for NSP, 9.81 and 8.00 for NGP, 1.13 and 0.69 g for TGW, 3.25 and 2.22 g for GY, explaining 31.03% and 25.89%, 16.79% and 7.39%, 24.11% and 16.15%, 52.49% and 10.95%, and 35.06% and 9.12% of the phenotypic variances, respectively. In the 14LS trial, the additive effect of *Hd1* and *Hd2* was 3.05 and 2.03 cm for PH, 9.42 and 8.11 for NSP, 13.07 and 9.76 for NGP, 0.88 and 0.36 g for TGW, 3.47 and 2.90 g for GY, explaining 32.23% and 16.93%, 27.90% and 20.55%, 38.27% and 18.12%, 54.42% and 2.83%, and 24.63% and 12.83% of the phenotypic variances, respectively.

**Fig. 5.**
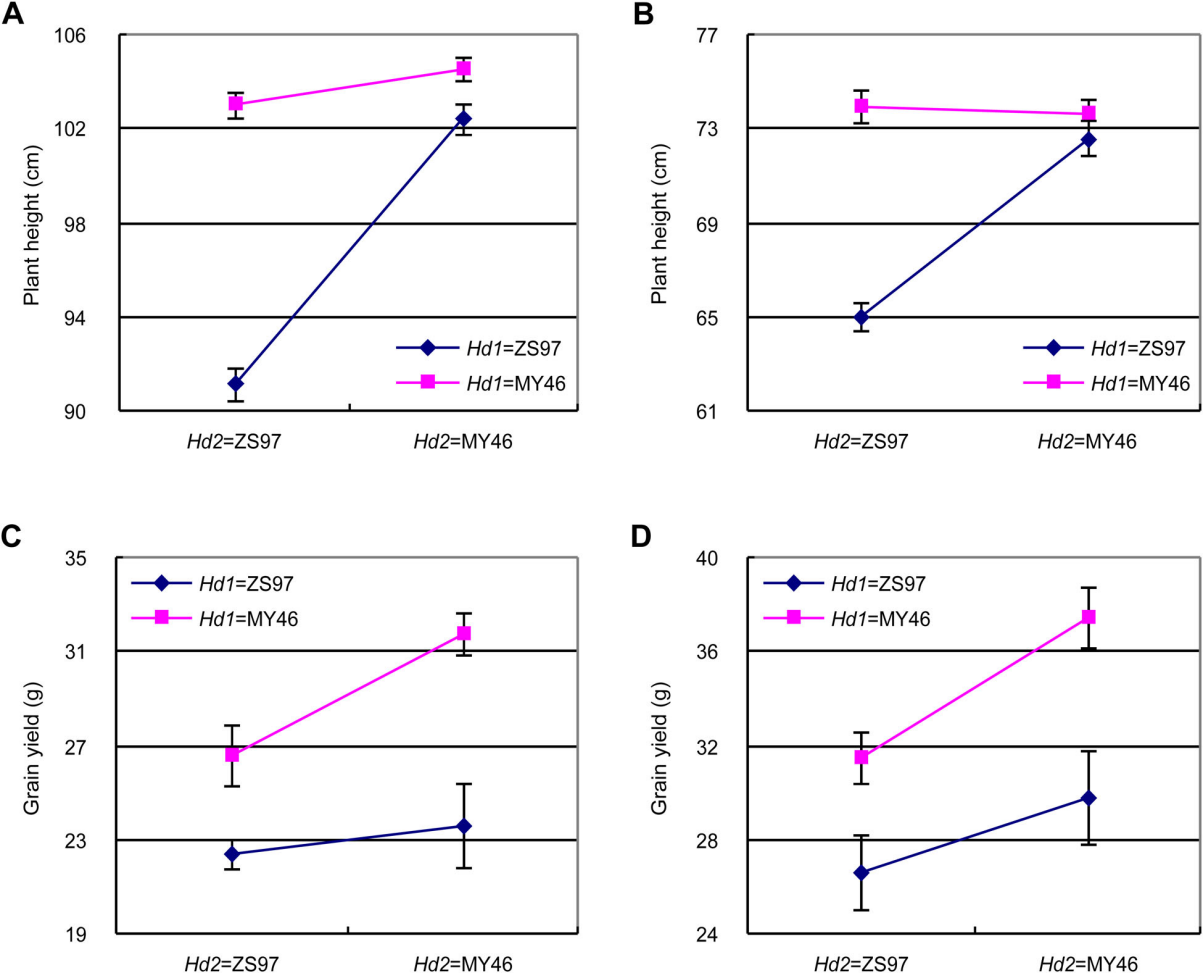
**Plant height and grain yield per plant of the four homozygous genotypic combinations of *Hd1* and *Hd2*.** (A,C) Under the NLD conditions in Hangzhou in the growing season of May−Sep in 2011, and (B,D) under the NSD conditions in Lingshui in the growing season of Dec 2013−Apr 2014. Data are represented as mean±s.e.m. *Hd1*^ZS97^*Hd2*^ZS97^, *n*=9; *Hd1*^ZS97^*Hd2*^MY46^, *n*=5; *Hd1*^MY46^*Hd2*^ZS97^, *n*=8; *Hd1*^MY46^*Hd2*^MY46^, *n*=9. Each line was tested in two replicates. In each replicate, eight plants were scored for plant height and five plants were measured for grain yield per plant.

In both trials, the two genes showed significant interaction for PH. The interaction acted for increasing the values of the recombinant types and its effect were 4.90 and 3.92 cm in the 11HZ and 14 LS trials, respectively (Table 3). The two recombinant genotypic groups, *Hd1*^ZS97^*Hd2*^MY46^ and *Hd1*^MY46^*Hd2*^ZS97^, were higher in PH than *Hd1*^ZS97^*Hd2*^ZS97^, but they were not significant different from *Hd1*^MY46^*Hd2*^MY46^ (Fig. 5A,B), indicating a duplicate action of the two genes in increasing PH in the NIL population analyzed in this study.

No significant interaction between the two genes were detected for any of the yield traits in the two trials despite that a marginal effect (*P*=0.0346) was observed for GY in the 11HZ trial. Similar to the significant digenic interaction detected for HD, the epistatic effect detected for GY was much smaller than the main effects of the two genes (Table 3; Fig. 5C,D), indicating that *Hd1* and *Hd2* also largely act additively for yield traits in the NIL populations analyzed in this study.

## DISCUSSION

### Interaction between *Hd1* and *Hd2* in controlling photoperiod response

Many flowering genes act together in controlling the photoperiod response in rice*.* Interactions between *Hd2* and other flowering genes were firstly reported by Yamamoto et al. (2000) and Lin et al. (2000), showing that this gene was interacted with *Hd1*, *Hd3* and *Hd6* but the interaction changed under different day-length conditions. Later, *Hd2* was also found to interact with other genes such as *Ghd7* and *Ghd8* (Fujino and Sekiguchi, 2005; Gu and Foley, 2007; Shibaya et al., 2011; Kim et al., 2013). Consequently, the response of *Hd2* to photoperiod varies depending on genetic backgrounds and environmental conditions. In the Nipponbare/Kasalath cross, it delayed flowering under LDs and promoted flowering under SDs (Yano et al., 2000). In the Zhenshan97/Teqing and the Kita-ake/PA64S crosses, *Hd2* delayed heading under LDs and had little effect under SDs (Liu et al., 2013; Yan et al., 2013; Gao et al., 2014). In our studies, photoperiod response of *Hd2* disappeared when the functional allele *Hd1*^ZS97^ showed no response to photoperiod in the background of ZS97, although the functional *Hd1*^ZS97^ and *Hd2*^MY46^ alleles continue to affect heading date by promoting and suppressing flowering, respectively, irrespective of the day-length conditions. Since populations used in this study and those showing no photoperiod response in the previous one (Zhang et al., 2012) were derived from the same BC_2_F_2_ plant, it can be assumed that the same factors required for the photoperiod response of *Hd1*^ZS97^ are eliminated in the ZS97 background. Subsequently, the photoperiod response of *Hd2*^MY46^ disappeared since *Hd1* is epistatic to *Hd2* in the genetic control of photoperiod response (Lin et al., 2000).

Among other flowering genes that interact with *Hd2*, *Ghd7* is a key factor involved in floral repression through *Hd1* (Nonoue et al., 2008; Nemoto et al., 2016). The activity of *Hd1* for repressing flowering under LDs disappeared in the absence of a functional *Ghd7* allele. The recurrent parent ZS97 used in our study was known to have lost a 38.3-kb fragment harboring the *Ghd7* gene (Xue et al., 2008). Using primer Se9153 (Table S1) for the *Ghd7-1* functional allele, no amplicon was produced from ZS97 (Fig. S3), but MY46 had a DNA fragment that was similar in size to the product of Minghui 63 which carries the functional allele *Ghd7-1*. Genomic DNA of *Ghd7* of MY46 was amplified using primers Ghd7sF and Ghd7sR (Table S1). Sequence analysis showed that the coding region of *Ghd7* in MY46 and Minghui 63 was identical (Fig. S4). In all the rice materials we used, the functional *Ghd7*^MY46^ allele has been removed based on marker genotyping of the *Ghd7* region. It could be concluded that the absence of functional *Ghd7* alleles is the reason why the functional *Hd1*^ZS97^ did not repress flowering under LDs.

Lin et al. (2000) found that *Hd1* is epistatic to *Hd2* under LDs but not under SDs. They also showed that the activity of *Hd2* for repressing flowering under LDs relied on the presence of functional *Hd1* allele. Since the genetic background of their populations was Nipponbare that carries a functional allele of *Ghd7* (Xue et al., 2008), the presence and absence of the Ghd7-Hd1 protein complex could be the reason for the interaction between *Hd1* and *Hd2* under LDs. In our studies, none of the rice materials carried functional *Ghd7* allele. In both the NSD and NLD conditions, the Ghd7 protein was not present, abrogating the interaction between *Hd1* and *Hd2*. These results suggest that the interaction between *Hd2* and *Hd1* essentially depends on the activity of *Hd1* for repression of flowering, in which *Ghd7* might play an important role*.* This is indeed shown in the 19 early-season *indica* rice varieties tested by Wei et al. (2012), of which eight and nine carry functional alleles at *Hd1* and *Ghd7*, respectively, but none of them carry functional alleles at both loci. The dependence of the *Hd1–Hd2* interaction on the presence of functional *Ghd7* allele was also confirmed in the original ZS97/MY46 RIL population when the RILs were grouped into eight sub-populations based on DNA markers linked to *Ghd7*, *Hd1* and *Hd2* (Fig. S5).

Yan et al. (2013) reported that the Teqing allele of *Hd2* in the ZS97 background repressed the expression of *Ehd1* and *Hd3a* under LDs but showed no effect under SDs. This not only differs from our observation that the PS activity of *Hd2* disappeared in the ZS97 background, but also fails to agree with the reversion of *Hd2* activity from delaying heading under LDs to promoting heading under SDs in other reports (Lin et al., 2000; Yamamoto et al., 2000). There are two possible reasons. First, only RM248 located in the *Hd2* region was used for selecting NILs used by Yan et al. (2013) and genotype information on other heading date genes were not clarified, it is possible that functional alleles at *Ghd7* or other loci that required for the PS activity of *Hd2* were preserved in the genetic background of the NILs they used. Second, *Hd2* has been found to interact with multiple genes (Lin et al., 2000; Yamamoto et al., 2000; Fujino and Sekiguchi, 2005; Gu and Foley, 2007; Shibaya et al., 2011; Kim et al., 2013), thus, its effects under LDs and SDs are highly diversified.

### Loss of photoperiod sensitivity of the functional alleles at *Hd1* and *Hd2* for adaption of rice to LDs in multiple season cropping

Natural variation in HD and PS has played a critical role in the wide adaption of rice to different cropping seasons and cultivation areas. Loss-of-function variants for genes controlling PS has been known to be the most important resource for *japonica* rice in northern cultivation regions to successfully reproduce under NLD conditions in a short summer period (Takahashi and Shimamoto, 2011; Wei et al., 2012; Fujino et al., 2013; Kim et al., 2013; Koo et al., 2013; Gómez-Ariza et al., 2015). At the same time, little knowledge has been accumulated for the genetic basis underlying the photoperiod-insensitive character of early-season *indica* rice cultivars in Southern China which also require successful reproduction under NLD conditions in summer thus sufficient time could be provided for late-season cropping. It is known that many early-season *indica* rice varieties grown in eastern or southern China carry functional alleles of *Hd1* (Wei et al., 2012) or *Hd2* (Yan et al., 2013). Among these varieties, ZS97 is an inbred variety and a maintainer line most commonly used in the three-line hybrid rice production in the 20th century. This variety carries the strong functional allele *Se-1^u^* at *Hd1* (Wei et al., 2012), but it is insensitive to photoperiod, presumably due to inhibiting factors in the ZS97 genetic background (Zhang et al., 2012). Analysis of the effects of PS genes in absence of their response to photoperiod will greatly broaden our understanding in the regulation of flowering time for wide adaption of rice cultivars.

HD of rice is divided into three phases: basic vegetative phase (BVP), photoperiod-sensitive phase (PSP), and reproductive phase (Nishida et al., 2001). As the durations of reproductive phase are similar among different varieties, differences of HD are determined by changes in BVP and PSP (Vergara and Chang, 1985; Nishida et al., 2001). Our experiments were conducted in both the NLD and NSD conditions, in which the effects of *Hd1* and *Hd2* were consistent across all the trials. On the other hand, all the NILs flowered much earlier in Hangzhou than in Lingshui (Fig. 4). These indicate that the effects measured for *Hd1* and *Hd2* are independent of the temperature and photoperiod differences between Hangzhou and Lingshui, whereas these differences strongly affected other flowering genes that were not segregated among the NILs. In other words, the PS of *Hd1* and *Hd2* were eliminated in our experiments, thus, their effects on HD could be ascribed to influence on BVP. The functional alleles *Hd1^ZS97^* and *Hd2*^MY46^ promoted and delayed heading under both the NSD and NLD conditions, respectively, which is accordance with previous reports in which the functional *Hd1* and *Hd2* alleles in Nipponbare controlled not only PS but also BVP (Nakagawa et al., 2005). The elimination of the PS activity of *Hd1* and *Hd2* assures that the *indica* rice varieties could flower under NLD conditions in summer, while a small range of HD variation is preserved duo to their effects on BVP.

In the last few years, an increasing number of genes/QTL for HD were reported to have pleiotropic effect on yield traits (Xue et al., 2008; Wei et al., 2010; Endo-Higashi and Izawa, 2011; Yan et al., 2011, 2013; Zhang et al., 2012; Liu et al., 2013; Zhao et al., 2013; Chen et al., 2014). However, most of them are involved in photoperiod response, contributing to yield enhancement accompanied with long growth duration under LDs (Xue et al., 2008; Wei et al., 2010; Endo-Higashi and Izawa, 2011; Yan et al., 2011, 2013; Liu et al., 2013). In the present study, pleiotropisms of *Hd2* and *Hd1* were tested when they had no response to photoperiod. The two genes showed consistent effects on plant height, grain number, grain weight and grain yield in both the NSD and NLD conditions, with the late-heading alleles always showing enhancing effects. It is also found that *Hd1* and *Hd2* largely acted additively in controlling yield traits with the removal of epistasis between the two genes due to the loss of PS activity of the functional *Hd1* allele. In conclusion, the presence of photoperiod-insensitive functional allele of *Hd1* and its influences on *Hd2* play an important role in grain yield and adaption of rice in multiple season-cropping systems.

## MATERIALS AND METHODS

### Construction of the rice populations

The rice populations used included seven NIL-F_2_ populations which were segregated in candidate regions for *qHD7.2*, including two populations in BC_2_F_6_ and five in BC_2_F_7_, and one set of NILs containing the four homozygous genotypic combinations of *Hd1* and *qHD7.2*/*Hd2*. The developing process was described below and illustrated in Fig. 1.

A residual heterozygote was identified from a BC_2_F_2_ population of the *indica* rice cross ZS97///ZS97//ZS97/MY46. This plant was heterozygous in intervals RM3438–Si9337 on the short arm of chromosome 6 and RM248–Se29626 on the distal end of the long arm of chromosome 7, which harbored HD genes *Hd1* and *qHD7.2*, respectively. The resultant BC_2_F_3_ population was assayed with DNA markers in the two target regions. One plant that was heterozygous in interval RM248−Se29626 and ZS97 homozygous in interval RM3438–Si9337 was selected. After two generations of selfing and marker genotyping, two BC_2_F_5_ plants retaining the RM248–Se29626 heterozygous segments were identified. Two BC_2_F_6_ populations were constructed and assayed with DNA markers in the RM248–Se29626 region. Five plants with overlapped heterozygous segments were selected and selfed to produce five BC_2_F_7_ populations, respectively (Fig. 2).

Other plants of the BC_2_F_3_ populations were harvested and selfed for three generations. The resultant BC_2_F_6_ population was assayed with DNA markers in the two target regions. A total of 31 non-recombinant homozygotes were identified, of which nine plants were ZS97 and MY46 homozygous for both *Hd1* and *Hd2*, respectively, five plants were ZS97 homozygous for *Hd1* and MY46 homozygous for *Hd2*, and eight plants were MY46 homozygous for *Hd1* and ZS97 homozygous for *Hd2*. These plants were selfed and a NIL set consisting of the four homozygous genotypic groups was constructed (Fig. 3).

### Field experiment

The rice populations were tested at experimental fields of the China National Rice Research Institute located either in Lingshui or Hangzhou (Table 1). In all the trials, the planting density was 16.7 cm between plants and 26.7 cm between rows. During the floral transition period of the rice materials, day lengths in Lingshui and Hangzhou corresponded to NSD and NLD conditions, respectively (Zhang et al., 2012). For the seven NIL-F_2_ populations, HD was recorded for each plant. For the NIL sets, the experiments followed a randomized complete block design with two replicates. For each replicate, eight plants per line were planted in one row. HD and PH were scored for each of the plants. At maturity, five middle plants in each row were harvested in bulk and measured for the four yield traits.

### DNA marker analysis

Total DNA was extracted following the method of Zheng et al. (1995). PCR amplification was performed according to Chen et al. (1997). The products of the DNA markers were visualized on 6% non-denaturing polyacrylamide gels using silver staining. All of the SSR markers were selected from the Gramene database (www.gramene.org). Primers for the three InDel markers, Si9337, Se29626 and Se29688 (Table S1), were designed using Oligo Primer Analysis Software Version 7.0 (Molecular Biology Insights, Inc.) based on InDels between ZS97 and MY46 detected by the whole-genome resequencing.

### Analysis of the coding sequence of *Hd2*

Total RNA was isolated from rice leaves using the RNeasy Plus Mini Kit (Qiagen, Japan) according to the manufacturer's instructions. The RNA was converted to cDNA using ReverTra Ace-α-™ (TOYOBO, Japan). The coding sequences of *Hd2* from the parents were amplified as two overlapping segments by PCR (KOD-plus neo, TOYOBO, Japan). The primers used for sequencing, Hd2s1 and Hd2s2 (Table S1), were designed using Oligo Primer Analysis Software Version 7.0.

### Data analysis

Linkage map construction and QTL analysis were performed for the two BC_2_F_6_ and three BC_2_F_7_ populations which were segregated for *qHD7.2*, including G6021, G6022, G7016, G7017 and G7018. The maps were constructed using Mapmaker/Exp 3.0 (Lander et al., 1987). Distances between markers were presented in centiMorgan (cM) derived using Kosambi function. QTLs were determined with the interval mapping of Windows QTL Cartographer 2.5 (Wang et al., 2012). For the NILs, two-way ANOVA was conducted to test the main and digenic interaction effect between the two loci. The analysis was performed with SAS procedure GLM (SAS Institute Inc., 1999).
